# Cellophane Banding Without Intraoperative Attenuation of Congenital Gastrophrenic Shunts in 12 Cases

**DOI:** 10.3390/vetsci12030190

**Published:** 2025-02-20

**Authors:** Martin Hamon, Philippe P. Haudiquet, Aurelie Bruwier, Kevin Schreiber, Renaud Jossier, Morgane Charbonneau, Pierre P. Picavet

**Affiliations:** 1 Department of Clinical Sciences, College of Veterinary Medicine, Kansas State University, Manhattan, KS 66506, USA; abruwier@vet.k-state.edu (A.B.); ppicavet@vet.k-state.edu (P.P.P.); 2 VetRef-Anicura, Referral Veterinary Clinic, 49070 Beaucouze, France; dr.haudiquet@orange.fr (P.P.H.); k.schreiber1@outlook.fr (K.S.); dr.charbonneau@yahoo.fr (M.C.); 3 VetSlice, 49240 Avrille, France; dr.jossier@gmail.com

**Keywords:** portosystemic shunt, gastrophrenic shunt, cellophane band attenuation, surgery, small animals

## Abstract

Reports on patients with a gastrophrenic shunt treated with cellophane banding without attenuation are scarce. This case series evaluated the outcomes of cellophane banding without intraoperative attenuation in patients with a congenital left gastrophrenic shunt. Seven client-owned dogs and five client-owned cats with congenital left gastrophrenic shunt were included. Data collected from the medical records included signalment, history, physical examination, clinicopathologic testing, diagnostic imaging (pre and postoperative), perioperative complications, hepatic histopathological evaluation, and clinical outcomes. The application of a cellophane band without intraoperative attenuation of congenital left gastrophrenic shunts resulted in complete closure in 10/11 patients at a median follow-up of 60 days (range: 33–174) based on ultrasound. The mean shunt diameter was 6.1 mm (range: 4.2–8). One dog had partial closure of the shunt at 3 months but was lost to follow-up. One dog died perioperatively from seizures. One cat experienced post-attenuation neurologic signs that completely resolved. Cellophane banding without intraoperative attenuation appeared to be a safe and potentially effective approach for managing gastrophrenic shunts. The percentage of shunt closure observed in this cases series is higher than that historically reported for other shunt localizations. Shunt localization may influence closure. Residual shunting may not be associated with shunt diameter. Further studies with larger sample sizes and standardized follow-ups are needed to confirm its efficacy.

## Simple Summary

Several techniques for gradual vascular occlusion have been described for the treatment of a congenital extrahepatic CPS, including cellophane banding. Residual shunting has been reported to be more common with cellophane banding than with other techniques, being 35% when no initial attenuation of the shunt was performed. This case series evaluated the outcomes and complications of 12 patients with a congenital left gastrophrenic shunt who were treated with cellophane banding without intraoperative attenuation. Complete closure of the shunt was observed on ultrasound in 10/11 patients at a median follow-up of 60 days. The clinical outcomes were excellent. This cases series suggests that cellophane banding without intraoperative attenuation may be a safe and effective technique for gastrophrenic shunts.

## 1. Introduction

Congenital portosystemic shunts (CPSs) are vascular anomalies providing direct communication between portal and systemic venous circulation by bypassing the liver [1]. Toy and small-breed dogs are generally affected by extrahepatic CPSs, whereas large and giant-breed dogs most commonly have intrahepatic CPSs. CPS is also observed in cats, with a much lower incidence [2]. The clinical signs associated with CPS involve the nervous system, gastrointestinal tract, and urinary tract [2]. Medical management improves clinical signs associated with CPSs, but surgical management results in better long-term survival [3].

In dogs, the most common extrahepatic CPSs are splenocaval, left gastrophrenic, left gastroazygos, and those involving the right gastric vein [4]. In cats, the most common are splenocaval, left gastrophrenic, left gastrocaval, and left gastroazygos [4]. Splenophrenic shunts have been described, but, according to the latest classification, they correspond to left gastrophrenic shunts. The specificity of left gastrophrenic shunt is its termination. It drains into the caudal vena cava caudally to the diaphragm and cranially to the liver [5].

Acute complete attenuation of the shunt at surgery often causes intolerable portal hypertension. Several techniques for gradual vascular occlusion have been described for the treatment of congenital extrahepatic CPSs, including partial ligation with a silk suture, ameroid ring constrictor, thin-film banding, intravascular thrombogenic coils, and hydraulic occluder [6,7,8]. Ameroid constrictors have an inner ring of hygroscopic substance (casein) that is surrounded by a stainless-steel sheath. Casein slowly absorbs body fluids and swells, reducing the internal luminal area [6]. Intravascular thrombogenic coils occlude the vessel lumen because a thrombus develops on and around the embolizing materials [6]. A hydraulic occluder consists of a cuff placed around the shunt. The inflation can be controlled percutaneously through injections of fluid into a subcutaneous injection port that is attached to the cuff by a length of actuating tubing [6]. The placement of a thin-film band around the CPS produces perivascular fibrosis responsible for the progressive vascular occlusion [9]. One study reported that 3.0 mm was the maximum internal diameter of the cellophane band (CB) applied at the level of the shunt that might be effective in producing complete closure [10]. Another study showed that intraoperative attenuation of the CPS to a diameter < 3.0 mm is not necessary and may result in a less favorable outcome with the possible development of acquired portosystemic shunts [11]. However, no imaging follow-up was performed in the latter study.

Residual shunting has been reported to be more common with cellophane banding than with other techniques, being 17% in one study [7] with partial attenuation and up to 31.6–35% when no intraoperative attenuation of the shunt was performed [1,5].

No studies have yet investigated the percentage of shunt closure according to the localization. Reports on patients with gastrophrenic shunt treated with cellophane banding without attenuation are scarce [5,11,12]. We hypothesized that the occlusion rate of gastrophrenic shunts treated with cellophane banding is high and that the occlusion can be achieved even with large-diameter shunts.

The objective of this case series is to report the outcome and complications of cellophane banding without intraoperative attenuation in patients with a congenital left gastrophrenic shunt.

## 2. Materials and Methods

### 2.1. Medical Record Data

The medical records of all dogs and cats diagnosed with an extrahepatic CPS between 2014 and 2023 at a single referral center were reviewed. The dogs and cats were included in this case series if a single congenital left gastrophrenic shunt was identified and attenuated with a cellophane band. The data collected included signalment, history, physical examination, clinicopathologic testing, preoperative and postoperative medications, preoperative and postoperative diagnostic imaging, perioperative complications, hepatic histopathological evaluation, and clinical outcomes.

### 2.2. Surgical Attenuation

The diagnosis of the left gastrophrenic shunt was obtained preoperatively either with advanced imaging (CT scan) or ultrasonography.

Preoperative medical management was prescribed for at least two weeks to decrease the occurrence of neurological symptoms. All the patients received at least 2 weeks of medical management before surgery with lactulose (0.5 mL/kg every 12 h PO), metronidazole (15 mg/kg every 12 h PO), and a prescription of liver or kidney diet. Levetiracetam (20 mg/kg every 8 h PO) was started 24 h before the surgery in 1 dog.

The same anesthetic protocol was applied for every patient. Premedication was performed with diazepam (0.2 mg/kg IV, Midazolam^®^, Mylan, Luitre, France) combined with morphine (0.2 mg/kg, IV in cats; 0.5 mg/kg, IV in dogs; morphine chlorhydrate Cooper^®^; Sanofi, Melun, France). Induction was obtained using propofol (4 mg/kg IV, titrated to effect; Propovet Multidose^®^; Zoetis, Louvain-La-Neuve, Belgium). An endotracheal tube was placed, and general anesthesia was maintained with isoflurane (Isorane^®^; Axience, Pantin, France) in dioxygen.

**Figure 1:**
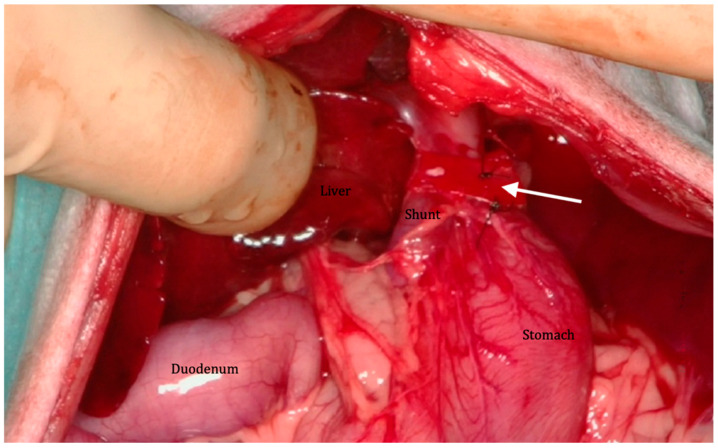
Intraoperative view of the cellophane band surrounding the gastrophrenic shunt (arrow).

All the surgical procedures were performed by a board-certified surgeon (MH or PH). After the exploratory celiotomy, the liver was retracted to the right and the stomach caudally. The shunt was identified because it traveled on the ventral surface of the abdominal portion of the esophagus before termination on the phrenic vein. The shunt vessel was dissected before it entered the diaphragm. The cellophane band (cellophane from commercially available suture packages’ wrapping: 10 cm long, 1.2 cm wide) was folded longitudinally to form a three-layered strip (10 cm long, 4 mm wide) and placed around the vessel using right-angle forceps. The band was positioned in complete contact with the CPS and then secured with 2 vascular clips (medium size; Peters Surgical, Boulogne-Billancourt, France) or 2 modified transfixion sutures with nylon (Ethilon^®^ 3-0; Ethicon, Issy les Moulineaux, France) (Figure 1). No intraoperative attenuation was applied at the level of the shunt. A liver biopsy was systematically performed.

Portal venous pressures were not measured during the surgical procedure, but indirect signs of portal hypertension (intestinal and pancreatic color changes and intestinal hypermotility) were monitored during placement of the cellophane band and before closure of the linea alba.

Postoperative management, including the use of analgesics (morphine 0.2 mg/kg, IV in cats; 0.5 mg/kg, IV in dogs), lactulose (0.5 mL/kg every 12 h PO), dietary protein restriction, antibiotics (metronidazole 15 mg/kg every 12 h PO), and anticonvulsants (levetiracetam 20 mg/kg every 8 h PO), was at the discretion of the surgeon and was recorded in each case. All the patients were monitored in hospital for 3 days.

### 2.3. Recheck Evaluation

At the time of reevaluation, an abdominal ultrasonography (associated with Doppler ultrasonography) was performed to assess shunt closure. A complete occlusion was concluded when a complete absence of Doppler flow was observed at the surgical site of occlusion. Direct and indirect signs of portal hypertension were also assessed. To exclude possible portal hypertension, the direction and velocity of portal flow were evaluated. A significant reduction in portal flow velocity or a hepatofugal flow are suggestive of portal hypertension. The presence of peritoneal effusion, gallbladder wall edema, pancreatic edema, and acquired shunts was also investigated.

If not performed by the referring veterinarian, a bile acid test was performed at the same time.

### 2.4. Data Analysis

The descriptive statistics were calculated in commercial software (XLSTAT 2017: Data analysis and statistics with Microsoft Excel; Addinsoft, Paris, France). The Shapiro–Wilk test for normality was performed for each variable (age, body weight, shunt diameter; postoperative serum bile acid concentration, and follow-up). The significance level was 0.05. The mean was calculated for normally distributed data, and the median was determined for nonnormally distributed data. Age, body weight, postoperative serum bile acid concentrations, and shunt diameter were data normally distributed. The follow-up was data nonnormally distributed (*p*-value = 0.011).

## 3. Results

### 3.1. Study Population

Seven dogs and five cats met our inclusion criteria (Supplementary Materials). The mean age was 34.6 months (range: 5 months–7 years). The mean age of the dogs was 45.6 months, and the mean age of the cats was 19.2 months. The overall mean body weight was 4.7 kg (range: 1.6–7.9). The mean body weight of the dogs was 5.8 kg (range: 3.1–7.9), and the mean body weight of the cats was 3.16 kg (range: 1.6–4.4). There were seven males and five females.

The canine patients’ breeds were Yorkshire Terrier (3), Shih Tzu (2), Maltese (1), and Poodle (1). The feline patients were one each of the following breeds: Birman, Maine Coon, Scottish Fold, Chartreux, and European Shorthair.

### 3.2. Shunt Diameter

**Figure 2:**
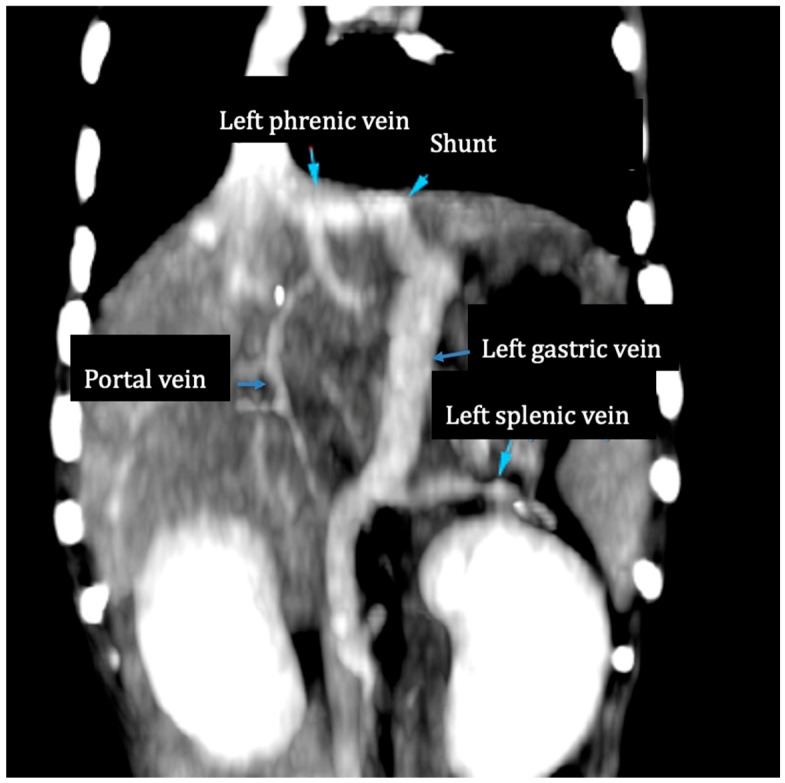
Post-contrast CT angiography. An enlarged left gastric vein joins the left phrenic vein at the level of the esophageal hiatus.

A preoperative CT angiography was performed in 11 patients (Figure 2). Abdominal ultrasonography was the only diagnostic exam available for one dog. The mean diameter of the shunts was 6.1 mm (range: 4.2–8).

Six cases (five dogs and one cat) had concurrent urolithiasis (kidneys and/or bladder stones).

### 3.3. Surgery

The cellophane band was successfully placed in all the patients. It was secured with two vascular clips (medium size; Peters Surgical, Boulogne-Billancourt, France) in one patient and two modified transfixion sutures using nylon (Ethilon^®^ 3-0; Ethicon, Issy les Moulineaux, France) in all the other patients. The surgery was performed successfully without intraoperative complications in any patients.

### 3.4. Results of Liver Biopsy

The histological changes were consistent with portal vein hypoperfusion or hypoplasia in all the patients.

### 3.5. Postoperative Medications

All the discharged patients were prescribed lactulose and a prescription liver or kidney diet until the recheck. Metronidazole was continued for 2 weeks after surgery in all the patients. Two cats received 1 month of levetiracetam postoperatively, and one dog received it for 2 weeks.

### 3.6. Complications

Two complications were reported (18%), and both were consistent with post-attenuation neurological signs (PANSs). One cat developed blindness and head pressing 3 days after the surgery, just a few hours after hospital discharge. The cat was initially treated with lactulose (0.5 mL/kg every 12 h PO), metronidazole (15 mg/kg every 12 h PO), and levetiracetam (20 mg/kg every 8 h PO). Amoxicillin–clavulanic acid (22 mg/kg every 12 h PO) was added. The head pressing resolved within 24 h. The blindness resolved spontaneously 3 weeks after the surgery. One dog developed seizures 2 days after the surgery. Midazolam (0.5 mg/kg IV) was administered. The seizure was unresponsive to treatment, so a propofol bolus (2 mg/kg IV) was administered, and the seizure stopped. The seizures recurred a few hours later, so the dog was placed on a propofol continuous-rate infusion. Levetiracetam was concurrently administered at 20 mg/kg every 8 h IV. The dog died from respiratory depression the following day.

### 3.7. Clinical Outcome and Evaluation of Shunt Closure

Of the remaining 11 patients, all were clinically normal at the time of recheck (median time of 60 days (range: 33–174)). Based on ultrasound at that time, ten had complete shunt closure characterized by the absence of Doppler flow at the surgical site of occlusion. In the last patient, an abdominal ultrasound showed a small residual flow at the level of the shunt at 3 months recheck. The vessel was smaller in size, but no measurement of shunt diameter was recorded. The dog was lost to follow-up. No acquired shunts were detected in any patient.

### 3.8. Postoperative Serum Bile Acid Measurement

The results of the analysis of the bile acid measurement in the postoperative period were available in nine patients. Pre- and postprandial bile acid concentrations were normalized in eight out of nine patients at the follow-up recheck. The mean of the preprandial bile acid concentration was 3.8 µmol/L (2.4–5.8), and the mean of the postoperative bile acid concentration was 8.3 µmol/L (6.4–9.8). The concentrations were still increased in the patient with the small residual flow (preprandial: 9.3 µmol/L; postprandial: 24.1 µmol/L).

## 4. Discussion

The application of a cellophane band to the congenital left gastrophrenic shunt without intraoperative attenuation resulted in complete closure in 10/11 patients based on ultrasonography. The clinical outcome was also excellent for these patients.

The advantages of cellophane banding include its ease of application and the low costs in comparison with other techniques [13]. Intraoperative portal pressure measurements are not necessary, and the duration of the surgery can, thus, be shortened [13]. Minimally invasive occlusion of CPSs with cellophane has been, therefore, increasingly described. One study described laparoscopic attenuation of 36 cases including 16 splenophrenic CPSs [14]. Partial or complete occlusion was systematically performed during the surgery, and all the shunts were closed after 2 months. A case report described the laparoscopic attenuation of a splenophrenic shunt (9.6 mm diameter) with a cellophane band, and no occlusion was applied at the level of the vessel [15]. Complete closure was also obtained. One hypothesis of the increased closure rate of this type of shunt is an increased perivascular fibrosis secondary to diaphragm motion with respiration cycle and gastric distension following meals. This specific localization might, therefore, be preferred for laparoscopic surgery.

A delayed closure has been described if the shunt diameter was more than 3 mm after cellophane band placement [10]. We obtained complete closure during our follow-up for a mean shunt diameter of 6.1 mm preoperatively. As no occlusion, even partial, was applied during surgery, the preoperative and immediate postoperative shunt diameter can be considered equivalent. However, the timing of postoperative ultrasonography has not been standardized, and it is, thus, possible that the closure occurred earlier in some patients.

Occlusion of the shunt should be performed as close to their insertion sites as possible to ensure redirection of the blood flow from all tributaries [5]. In gastrophrenic shunts, it is recommended to place the cellophane before the shunt enters the diaphragm [16]. Dissection at the level of the central tendon of the diaphragm is not easy, and it is common to dissect the shunt with the tendon fiber. It can cause occlusion failure despite an adequate inflammatory response [16].

Recanalization of a shunt with the use of cellophane banding has been described in a cat [17], and the issue of species differences in response to cellophane banding has been argued by some authors [18]. It is worth mentioning that this recanalization was described on a shunt that always had residual flow at follow-up, questioning the concept of “recanalization” itself. Complete closure of the CPS was observed in the five cats of the present study, and no apparent delayed closure was observed.

In the case report [17] in which a recanalization was observed in a cat, commercial roll cellophane and pure cellophane were used in the first and second attempts to close the shunt. Both types of cellophane resulted in vessel “recanalization”. The authors of the present study do not believe that the biochemical composition of the cellophane film used in the present study influenced successful closure. The authors used the same cellophane for other types of CPSs and had cases with residual shunting.

CT angiography has been used to assess the liver and vasculature after surgical attenuation [5]. CT angiography can also provide information about residual shunting or the development of acquired shunts [5]. In the present study, abdominal ultrasonography was used as the first follow-up imaging technique as it avoids general anesthesia and is less expensive for owners. In cases of doubt concerning residual shunting or the development of acquired shunts, a control CT angiography is generally performed, but ultrasonography was considered sufficient in our cases due to the absence of Doppler flow at the surgical site of occlusion. There is no study evaluating the sensitivity and specificity of ultrasonography for the detection of postoperative residual shunting [1].

The measurement of bile acids in the blood and the presence or absence of clinical signs have been regularly used as tools for the follow-up of patients with a CPS treated with cellophane banding and no occlusion [11,12]. However, no CT or abdominal ultrasound was performed in those studies to assess the closure rate. The lack of routine postoperative imaging means that some patients with persistent shunting were likely missed. Also, some animals can continue to present biochemical evidence of hepatic dysfunction despite closure of the original shunt [18].

Two patients developed PANSs. They were the older dog and the older cat. However, some studies report no significant difference in the age of dogs and cats that did or did not develop PANSs [19,20]. The idiopathic nature of PANSs precludes specific treatment. No consensus exists regarding their management. Supportive care and the management of neurologic signs are the only recommendations [19,20].

The limitations of our study include the small number of patients and the retrospective nature of this study. Further studies with larger sample sizes and standardized follow-ups are needed. A multi-institutional study could also compare the closure of gastrophrenic shunts with that of other types of shunts. It would be interesting to know whether the shunt location influences closure.

## 5. Conclusions

This case series suggests that cellophane banding without intraoperative attenuation may be a safe and effective technique for gastrophrenic shunt attenuation, with a closure in 91% of cases. Shunt localization may influence the closure because the percentage of shunt closure was higher than reported historically. Further studies with larger sample sizes and standardized follow-ups are needed to confirm its efficacy.

## Data Availability

Data available on request from the authors.

## Supplementary Materials

The following supporting information can be downloaded at: https://www.mdpi.com/article/10.3390/vetsci12030190/s1.

## Abbreviations

CPSs	Congenital portosystemic shunts
PANSs	Post-attenuation neurological signs
CT	Computed tomography
