# Supplementary material for: Cellophane Banding Without Intraoperative Attenuation of Congenital Gastrophrenic Shunts in 12 Cases
Source: Vet Sci. 2025 Feb 20;12(3):190. doi: 10.3390/vetsci12030190 (PMC11945643; doi:10.3390/vetsci12030190)
Supplement: Supplementary file 1 [file vetsci-12-00190-s001.zip › vetsci-3446977-supplementary.pdf]

Patient	Species	Breed	Age at the surgery	Diameter of the shunt (mm)	Preoperative medications	Postoperative medications	Postoperative complications	Time at the recheck (days)	Closure of the shunt on ultrasound (Yes; Y / No; N)	Postoperative serum bile acid measurement
1	Dog	Poodle	2y 10m	5.1	lactulose + metronidazole	lactulose + metronidazole		42	Y	Within normal limits
2	Dog	Maltese	1y 6m	4.3	lactulose + metronidazole + levetiracetam	lactulose + metronidazole + levetiracetam		66	Y	Within normal limits
3	Dog	Yorkshire Terrier	7y 7m	6.2	lactulose + metronidazole		Seizures, cardiorespiratory arrest			
4	Cat	Birman	5m	5.9	lactulose + metronidazole	lactulose + metronidazole + levetiracetam		40	Y	Within normal limits
5	Cat	European shorthair	3y 10m	6.1	lactulose + metronidazole	lactulose + metronidazole + levetiracetam + amoxycillin-clavulanic acid	Blindness and head pressing	70	Y	Within normal limits
6	Dog	Yorkshire terrier	5m	3.6	lactulose + metronidazole	lactulose + metronidazole		84	N	Abnormal
7	Cat	Maine Coon	8m	7.2	lactulose + metronidazole	lactulose + metronidazole		33	Y	Within normal limits
8	Dog	Yorkshire	3y 6m	7.6	lactulose + metronidazole	lactulose + metronidazole		174	Y	Within normal limits
9	Cat	Scottish fold	1y 7m	4.2	lactulose + metronidazole	lactulose + metronidazole		54	Y	Not available
10	Dog	Shih Tzu	6y 3m	8	lactulose + metronidazole	lactulose + metronidazole		140	Y	Within normal limits
11	Cat	Chartreux	1y 6m	8	lactulose + metronidazole	lactulose + metronidazole		68	Y	Within normal limits
12	Dog	Shih Tzu	4y 6m	6.5	lactulose + metronidazole	lactulose + metronidazole		49	Y	Not available
